# Inspection of Spot Welded Joints with the Use of the Ultrasonic Surface Wave

**DOI:** 10.3390/ma16217029

**Published:** 2023-11-03

**Authors:** Dariusz Ulbrich, Grzegorz Psuj, Artur Wypych, Dariusz Bartkowski, Aneta Bartkowska, Arkadiusz Stachowiak, Jakub Kowalczyk

**Affiliations:** 1 Faculty of Civil and Transport Engineering, Poznan University of Technology, 60-965 Poznan, Poland; arkadiusz.stachowiak@put.poznan.pl (A.S.); jakub.kowalczyk@put.poznan.pl (J.K.); 2 Center for Electromagnetic Fields Engineering and High-Frequency Techniques, Faculty of Electrical Engineering, West Pomeranian University of Technology in Szczecin, 70-313 Szczecin, Poland; gpsuj@zut.edu.pl; 3 Faculty of Materials Engineering and Technical Physics, Poznan University of Technology, 61-138 Poznan, Poland; artur.wypych@put.poznan.pl (A.W.); aneta.bartkowska@put.poznan.pl (A.B.); 4 Faculty of Mechanical Engineering, Poznan University of Technology, 61-138 Poznan, Poland; dariusz.bartkowski@put.poznan.pl

**Keywords:** ultrasound, spot welding, surface wave, car body, joint quality, FFT

## Abstract

Spot welded joints play a crucial role in the construction of modern automobiles, serving as a vital method for enhancing the structural integrity, strength, and durability of the vehicle body. Taking into account spot welding process in automotive bodies, numerous defects can arise, such as insufficient weld nugget diameter. It may have evident influence on vehicle operation or even contribute to accidents on the road. Hence, there is a need for non-invasive methods that allow to assess the quality of the spot welds without compromising their structural integrity and characteristics. Thus, this study describes a novel method for assessing spot welded joints using ultrasound technology. The usage of ultrasonic surface waves is the main component of the proposed advancement. The study employed ultrasonic transducers operating at a frequency of 10 MHz and a specially designed setup for testing various spot welded samples. The parameters of the spot welding procedure and the size of the weld nugget caused differences in the ultrasonic surface waveforms that were recorded during experiments. One of the indicators of weld quality was the amplitude of the ultrasonic pulse. For low quality spot welds, the amplitude amounted to around 25% of the maximum value when using single-sided transducers. Conversely, for high-quality welds an amplitude of 90% was achieved. Depending on the size of the weld nugget, a larger or smaller amount of wave energy is transferred, which results in a smaller or larger amplitude of the ultrasonic pulse. Comparable results were obtained when employing transducers on both sides of the tested joint, as an amplitude ranging from 13% for inferior welds to 97% for superior ones was observed. This research confirmed the feasibility of employing surface waves to assess the diameter of the weld nugget accurately.

## 1. Introduction

Joining vehicle body components is a significant technological challenge due to the need to ensure adequate body rigidity as well as the ability to absorb energy in road accidents. The methods of joining steel sheet elements used in motor vehicles include: welding, butt-welding [1], laser welding [2], spot welding [3,4], adhesive bonding [5,6], and riveting [7]. Spot welding is widely used in the automotive industry and has been used for many years, but it requires quality control at the manufacturing stage as there may be around 6000 spot welds on a vehicle body [8,9]. The spot welding method is not only used for joining steel components of vehicle bodies, but also allows joining element produced by additive manufacturing technology [10] with aluminum [3,11,12], which makes the technique promising in future construction development.

Among the methods for evaluation the quality of spot welded joints both, destructive and non-destructive methods can be distinguished [13,14]. The destructive methods measure the remaining weld nugget in two perpendicular directions and calculate the shear stress that destroys the joint. Non-destructive methods for testing spot welded joints include ultrasonic [15], radiographic [16], magnetic [17,18] and thermographic methods [19,20]. Vision systems for evaluating spot welded joints and machine learning methods applied during their manufacturing on the production line have also been proposed [21,22,23]. However, the ultrasonic method is the one that is the most commonly used to evaluate the spot welded joints among the non-destructive testing techniques discussed above.

Standard ultrasonic testing of spot welded joints of vehicle bodies is conducted using a longitudinal wave of 15–20 MHz [24]. In this case, the ultrasonic wave is generated and sent into the joint area by the ultrasonic head. The selection of the diameter of the ultrasonic transducer is made according to the thickness of the joined steel sheets, considering the smallest thickness to detect the minimum diameter of the weld nugget, which ensures the high strength of the spot welded joint. Surface irregularities occur due to material plasticization during the welding process, so ultrasonic heads use a rubber membrane that adjusts to the trace created by the pressure of the electrodes during the spot welding process [25]. These heads also have a water delay. Before the ultrasonic wave begins to propagate in the spot welded connection, it travels a distance in the water, allowing for the measurements outside the dead zone and the near field. However, the disadvantage of this method is the need to apply the ultrasonic head at the site of the joint, which is easy to perform during the production stage but is hindered by the installed components of the vehicle during the operational stage. The main result of the study is the waveform of ultrasonic longitudinal wave pulses reflected from the bottom of the joint. Characteristic ultrasonic waveforms for properly made welded joints and those with defects are summarized in Ref. [26]. Based on the echo sequences, high-quality joints, weld nuggets with a too small diameter, sticking, and complete failure to connect the welded sheets were identified, each characterized by a different course of echoes, including the possible occurrence of intermediate echoes.

Another, more modern testing technique than the one described above is the use of the Phased Array method [27,28]. This method allows a C-Scan measurement of the weld nugget on the screen of the ultrasonic flaw detector, as opposed to standard A-Scan testing, which only displays the course of ultrasonic wave pulses reflected from the bottom of the spot welded joint.

There are also other techniques for testing spot welded joints using the ultrasonic method, however they are generally utilized in laboratories and are rarely employed under industry conditions. For example, Lamb waves are used to estimate the size of the weld nugget. Bendec et al. [29] proved that the transmission coefficient of the Lamb wave is proportional to the cube of the weld diameter, and the determination of the welded area is based on changes in the amplitude of the ultrasonic wave. The Lamb wave was also used by Takada et al. to evaluate the quality of spot welded joints [30]. In their study it was shown that the nugget diameter of a spot weld can be evaluated using the system built by them along with a through-transmitted wave. The results show that the diameter of the weld nugget can be assessed by measuring the width of the zone where the through-transmitted wave is significantly weakened. Xiaokai et al. [31] performed both ultrasonic and mechanical tests to determine the maximum tensile-shear strength. Using the PSO-SVM (particle swarm optimization support vector machine) and BP (back-propagation) classifier, the possibility of correlating the ultrasonic parameters of the longitudinal wave in the time and frequency domain with the mechanical strength of the spot welded joint was determined.

Although, as it was presented, the ultrasonic technique offers promising possibilities for obtaining satisfactory results for testing spot welded joints, there remains an area combining proposals for testing procedures with the difficulties of measuring real construction joint elements. The main goal of the research was to propose a new approach in testing spot welded joints. Estimation of the nugget diameter of a spot weld used in the construction of motor vehicles by surface wave was proposed. The scientific aim was to determine the differences in waveforms of the ultrasonic surface wave, which propagates through the spot weld for joints made with different welding parameters as well as FFT (Fast Fourier Transform) obtained for two different measurements (stage 1 and stage 2). A novel aspect of the proposed procedure for testing spot welded joints is the passage of the surface wave through the spot welded joint area and its reception on the other side of the steel plate, as well as the application of the ultrasonic surface wave, also known as a Rayleigh wave (stage I). In the second stage of the research, a surface wave through the welded joint was transmitted (the ultrasonic heads were placed on the same side of the welded joint).

## 2. Materials and Methods

**Figure 1:**
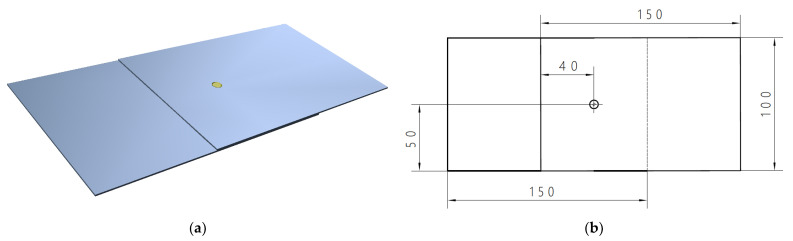
Sample with spot weld prepared for ultrasonic testing; (**a**) 3D model and (**b**) 2D model with the dimensions.

Ultrasonic testing of non-separable joints was carried out on steel plate samples that had been spot welded with an overlap. Steel sheets obtained from vehicle body parts and specimens of 100 mm × 150 mm × 0.8 mm were prepared. The spot welding was performed in the center of the specimen. A view of the sample after the joining process is shown in Figure 1. Twelve specimens were used for the test. The samples were prepared with different welding process parameters. This resulted in different diameters of weld nugget, so the strength and quality of the spot welded joint was changed. The spot welding process parameters for each sample used during the study are summarized in Table 1. The parameters for the production of spot welded joints were selected based on the available literature [32,33] and the possibility of changing the parameters of the resistance spot welding process. Taking this into account, samples with different weld nugget diameters were prepared, which clearly correlated with the quality of the joint.

The samples were made using an RZP 2A welding machine (Figel, Gdanska, Poland). The constant pressure was 200 N. In addition, two parameters of the process were changed: the welding current and the time of the spot welding process. Three different welding current values were used, where designation 2 is the lowest welding current value and 4 is the highest. The spot welding time was also modified and ranged from 0.15 to 1.5 s. As a result, 12 spot welded joints were obtained, which had different quality due to the above process parameters for joining body sheets.

In the next stage of experiment, the samples were subjected to ultrasonic surface wave testing. Ultrasonic tests were carried out in two stages:

- Tests using surface wave heads placed on different sides of the spot welded joint (stage I);
- Tests using surface wave heads placed on the same side of the spot welded joint (stage II).

**Figure 2:**
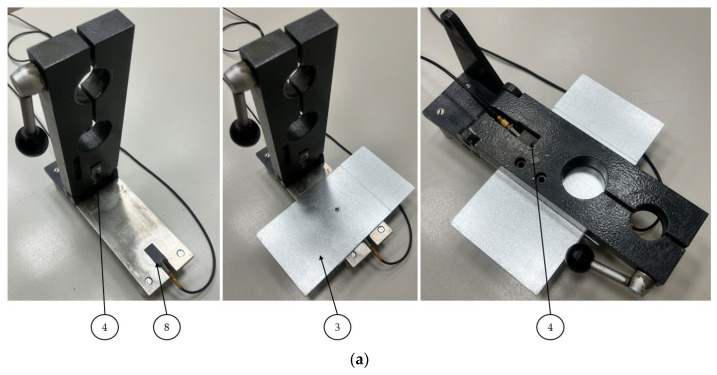

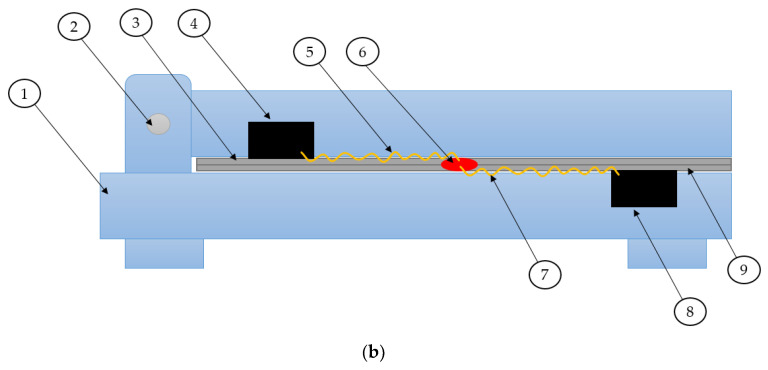
A specially designed holder for stage I (**a**) and the scheme of the ultrasonic measurement at different sides (**b**) of the spot welded joint: 1—holder, 2—pivot point of the upper arm, 3—steel plate 1, 4—ultrasonic transmitting head, 5—ultrasonic wave on the steel plate 1, 6—weld nugget, 7—ultrasonic wave on the steel plate 2, 8—ultrasonic receiving head, and 9—steel plate 2.

For stage I of the ultrasonic experiment, a specially designed holder was prepared (Figure 2a), which allowed us to mount two ultrasonic transducers and place the sample between them. In addition, the upper arm of the holder can be lifted up along with the ultrasonic transmitting head, which allowed us to perform several ultrasonic measurements of spot weld without changing the position of the sample. It was necessary to use two Karl Deutsch S6 WB 10 WM (Karl Deutsch, Wuppertal, Germany) ultrasound heads that operate at a frequency of 10 MHz and generate a longitudinal wave, as well as wedges specifically designed for the heads that refract the longitudinal wave at a 90-degree angle to produce a surface wave. The ultrasonic heads were permanently mounted on a holder, and a view of their positioning in relation to the sample is shown in Figure 2b. Nondestructive testing was carried out using the pass-through technique, where one head generates an ultrasonic wave pulse and the other receives it. Ultrasonic tests using the UMT 15 flaw detector (Ultra, Radom, Poland) were carried out.

The important thing is that the ultrasonic wave propagates along the surface of the first sheet, then passes through the weld nugget and is received on the other side of the second sheet, where the receiving head was mounted (Figure 2b). For each of the 12 specimens, 5 ultrasonic measurements of the wave propagating through the spot welded joint were made. During the measurements the specimen and heads positioned unchanged. The pulse waveform was recorded in the time domain. Ultrasonic measurements were made at a wave frequency of 10 MHz. The gain of the wave pulse was 50.00 dB. In the next stage, the observation period was set to 127.60 us. In the final stage, the transmitter voltage was set to 170 V, and the bandwidth was set to 200 ns, which allowed us to obtain a stable pulse from the area of all tested spot welded joints.

**Figure 3:**
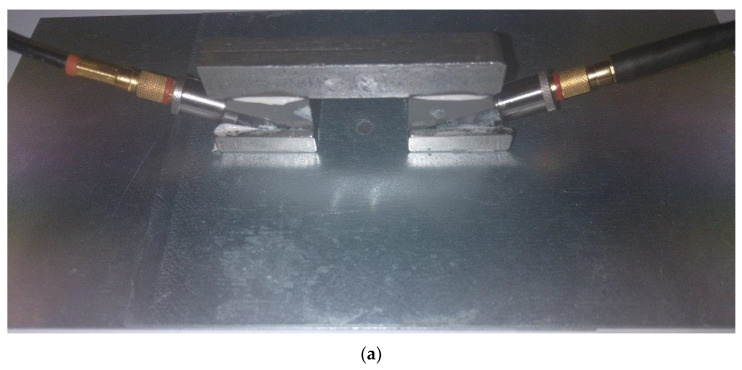

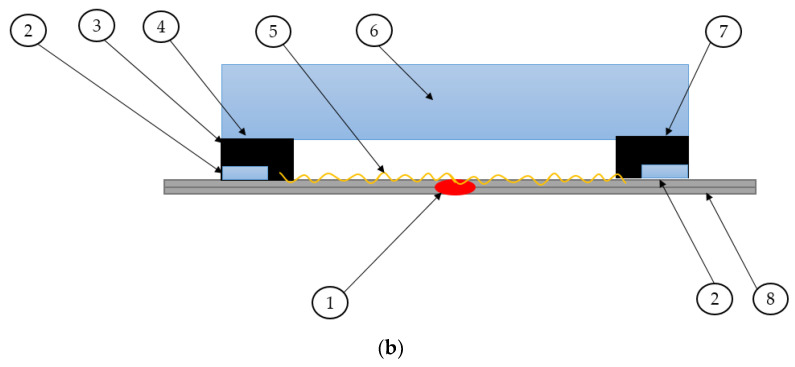
A specially designed holder for stage II (**a**) and the scheme of the ultrasonic measurement on the same side of the sample (**b**) of the spot welded joint: 1—spot weld, 2—magnets, 3—ultrasonic transmitting head 4—the place where the strip is glued to the ultrasonic head, 5—ultrasonic wave on the steel plate, 6—mounting strip, 7—ultrasonic receiving head, and 8—steel plates.

The second stage of ultrasonic measurements included testing of spot welds, in which the ultrasonic wave was sent and received using heads placed on the same side of the spot weld (Figure 3). The parameters of the emitted surface wave were identical to those in the first stage of ultrasonic testing, and 5 ultrasonic measurements were performed for each spot weld. The ultrasonic heads were bonded to a steel strip (ensuring a constant distance between the heads), and additionally, in order to ensure constant pressure to the sample, the ultrasonic heads had bonded neodymium magnets.

After the realization of ultrasonic measurements, shear force tests of the welded joints were performed. The tests were realized on a Cometech B1/E testing machine (Cometech testing machines, Taichung City, Taiwan), which allowed axial mounting of the specimens (joints were installed) and recording of the maximum destruction force of the joint. The jaw speed in the test was equal 2 mm/min. The machine had a measuring range of 15 kN. In addition, the analysis of the residual weld nugget was carried out, and the measurement of the diameter of the nugget in two perpendicular directions was performed.

The final stage of the study searched for correlations between the parameters of the ultrasonic wave pulse propagating through the joint and the destructive force of the spot weld.

## 3. Results

**Figure 4:**
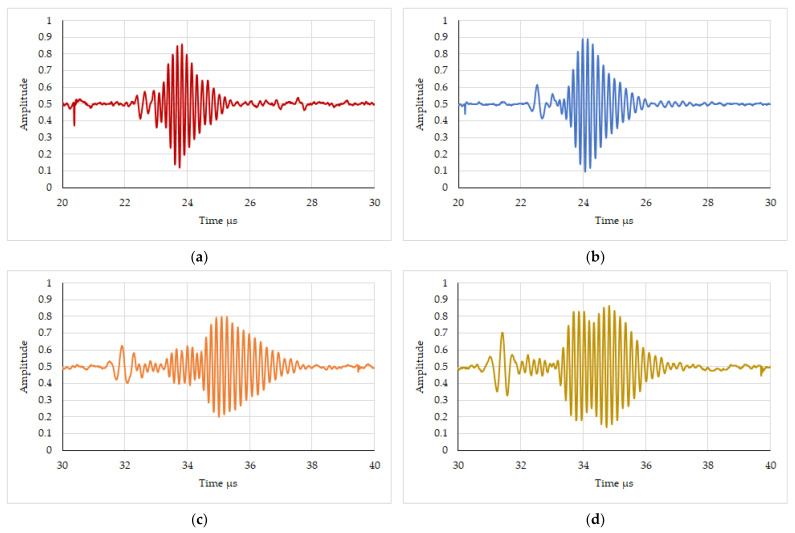
Result of ultrasonic testing of spot welds. (**a**,**b**) Test for different spot welding process parameters—heads mounted on the same side of the spot weld. (**c**,**d**) Test for different spot welding process parameters—heads mounted on the different side of the spot weld.

The selected sample results of ultrasonic testing of spot welded joints for each specimen in the time domain are shown in Figure 4 (Figure 4a,b show the heads mounted on the same side of the sample and Figure 4c,d show the heads mounted on different sides of the spot weld). These are exemplary pulse waveforms of a surface wave propagating through a spot welded joint. It is clear from the waveforms that, depending on the parameters of the spot welding process, different values of the wave transit time and their amplitude were obtained. It can also be seen that the surface wave transposes and for some samples two pulses are visible from the area of the tested connection. This was clearly noticeable in the case of testing with ultrasonic heads placed on different sides of the spot welded joint. For time domain tests, the maximum pulse height of the surface ultrasonic wave was recorded as a parameter that varies depending on the quality of the spot welded joint.

**Figure 5:**
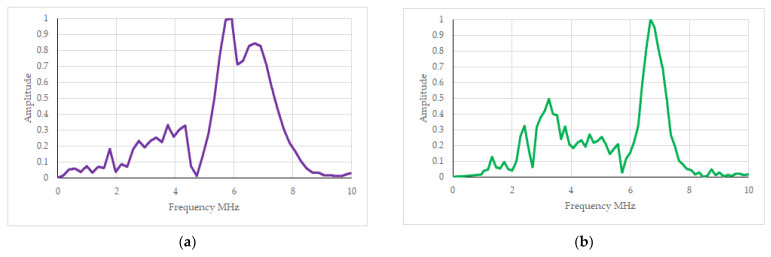

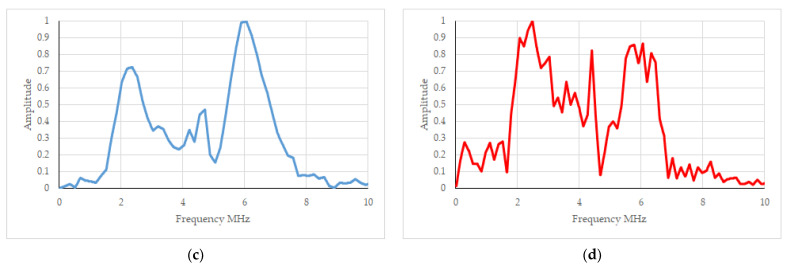
Results of ultrasonic tests in the frequency domain, represented as FFT envelopes for various samples. (**a**,**b**) Test for different spot welding process parameters with heads mounted on the same side of the spot weld. (**c**,**d**) Test for different spot welding process parameters with heads mounted on different sides of the spot weld.

The analysis was also carried out using the fast Fourier transform. This allowed us to record ultrasonic wave parameters such as the pulse envelope in the frequency domain. The frequency-domain ultrasonic measure was the frequency at the maximum amplitude of the FFT spectrum. Exemplary results of the envelope of the amplitude-frequency spectrum are shown in Figure 5. When testing with heads placed on the same side of the spot welded joint (stage II), one maximum frequency is always visible, which ranged from approximately 6 to 8 MHz. However, when testing the spot welded joint with heads mounted on both sides of a steel sheet (stage I), two frequency peaks and significant changes in the band between these peaks are always visible.

Detailed ultrasonic test results for all samples are presented in Table 2, Table 3, Table 4 and Table 5. The tables include the results of tests in the field of time and frequency domain (5 measurements each), with the average values of the selected parameters as well as standard deviation and measurement error.

**Figure 6:**
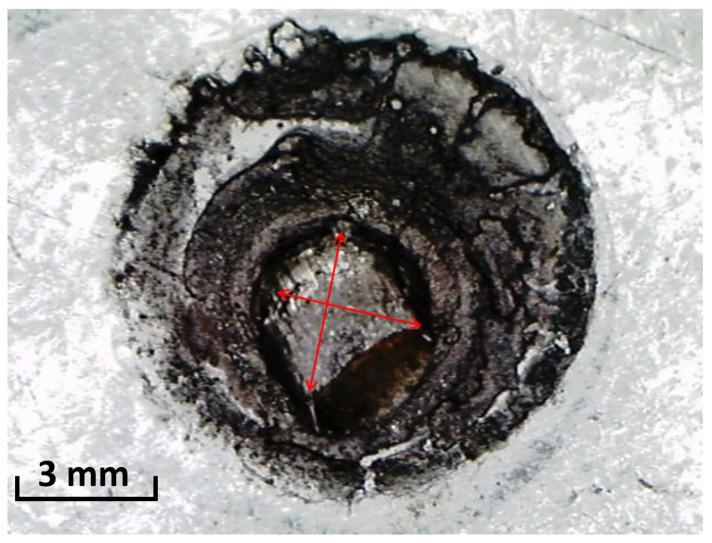
The principle of measuring the diameter of the weld nugget after the destructive test of car body sheets. The red arrows mark the locations of the spot weld nugget measurements.

Next, to complete the analysis, the destructive tests were performed. The shear test of the spot welded joint allowed us to determine the force that destroyed the joint and to determine the remains of the weld nugget. The results of these tests were presented in Figure 6 and Table 6. The tests of the spot weld nugget were carried out in two perpendicular directions in order to estimate the average value of this parameter. In the case of the results of the shear test, there is also a relationship between the process parameters and the value of the force that destroys the joint. The time of the spot welding process significantly affects the diameter of the weld nugget. The longer this time is (even with the same parameters of pressure and welding current), the larger the weld nugget and the higher the value of the shear force needed to destroy the spot welded joint. In addition, there is some irregularity of the weld nugget, with the diameter typically being higher in one of the measured directions than in the other. This is due to the fact that the process of spot welding of samples was performed manually (not using a station with robots). The maximum value of the destructive force of the welded joint was over 6 kN (samples 4_4 and 4_5) and was more than six times higher than the lowest force value recorded for samples 2_1. In addition, a slight increase in weld nugget diameter is noticeable depending on the welding current setting. In the case of five samples, kissing bond (stick weld) was found, i.e., no diameter of the weld nugget. These are low quality connections. Nevertheless, although the diameter of the weld nugget was not created, the ultrasonic wave propagated through the joint area and it was possible to record important information from the point of view of the weld quality.

## 4. Discussion

**Figure 7:**
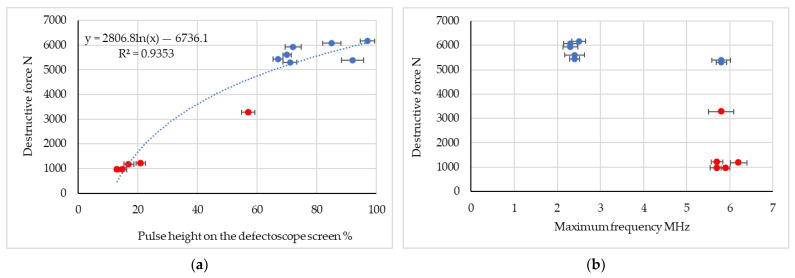
Ultrasonic test results for testing with ultrasonic heads on different sides of the spot- welded joint (red dot—kissing bond—low quality joint, blue dot—good quality joint); (**a**) correlation of the destructive force with the maximum amplitude (ultrasonic pulse height) for all spot welded joints, (**b**) correlation of the maximum frequency of the Fourier spectrum with the force destroying the spot welded joint.

**Figure 8:**
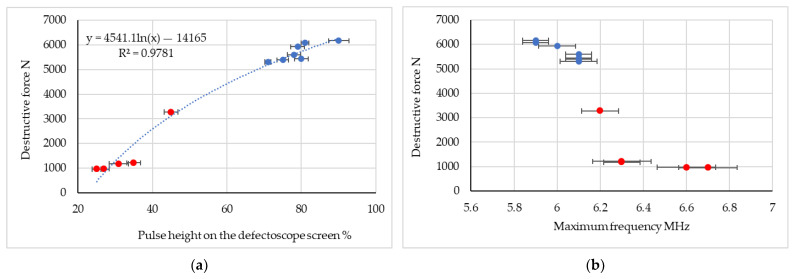
Ultrasonic test results for testing with ultrasonic heads on the same sides of the spot- welded joint (red dot—kissing bond (low quality joint), blue dot—good quality joint); (**a**) correlation of the destructive force with the maximum amplitude (ultrasonic pulse height) for all spot welded joints, (**b**) correlation of the maximum frequency of the Fourier spectrum with the force destroying the spot welded joint.

The average results from each measurement of ultrasonic pulse waveforms depending on the spot welded joint (manufacturing parameters) are shown in Figure 7 and Figure 8. The results are presented in the form of a summary of the pulse height (amplitude) and the maximum frequency of the amplitude-frequency spectrum depending on the force destroying the spot welded connection. The research stages related to the method of mounting the ultrasonic heads were also taken into account.

The research results showed that when using an ultrasonic surface wave there is no need for direct access to the surface of the spot weld, because the surface wave heads are placed at a certain distance from the spot weld joint. This allows measurement in post-failure or post-accident inspection conditions, as opposed to the classic method, where research is carried out mainly during the production stage. The advantage of the developed method is the ability to conduct research in the frequency domain. Conducting research in the frequency domain is important because the results of ultrasonic tests are not influenced by the head pressure force and the amplification of the ultrasonic pulse. For all tests, good quality connections (the blue dots in Figure 7 and Figure 8) as well as kissing bonds (the red dots in Figure 7 and Figure 8) were identified. Significantly more accurate results in the frequency domain for heads placed on the same side of the connection (Figure 8b) were detected. In the case of stage II, it was clearly stated that the lower the value of the maximum frequency of the amplitude-frequency spectrum, the higher the force necessary to destroy the spot welded joint. Therefore, it can be concluded that the lower the value of this frequency, the higher the quality of the spot welded joint.

**Figure 9:**
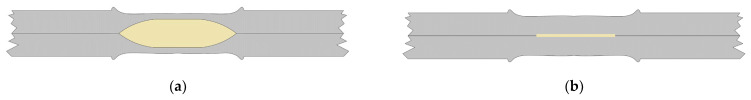
Cross-sectional diagram of the weld nugget of high quality (**a**) and low quality kissing bond (**b**).

**Figure 10:**
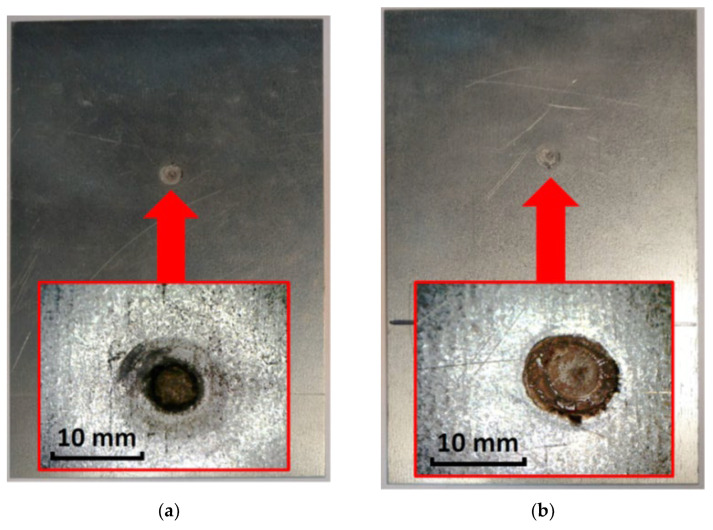

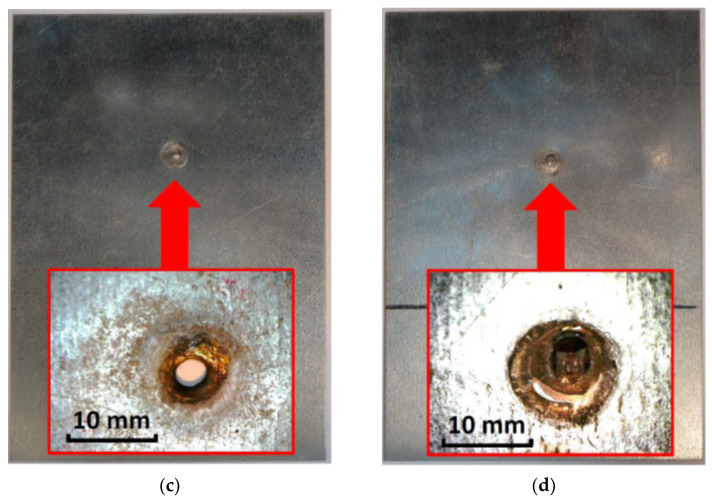
View of two samples after the destructive process of the welded joint: (**a**) sample with low quality of the welded joint, sheet I, (**b**) sample with low quality of the welded joint, sheet II, (**c**) sample with high quality of the welded joint, sheet I, (**d**) sample with high quality of the welded connection, sheet II.

In the case of assessing the quality of the spot weld using the amplitude of ultrasonic pulses, important results were obtained for both ways of applying the ultrasonic heads (Figure 7a and Figure 8a). For low-quality connections (kissing bond), regardless of the place of installation of the ultrasonic heads (stage I and stage II), it was shown that the amplitude of the ultrasonic pulse ranges from approximately 13 to 35% of the height of the flaw detector screen (maximum pulse). For higher quality connections, higher values of the ultrasonic surface wave pulse amplitude were detected. The research results confirm that it is possible to use relatively simple and cheap head mounting systems. This allows for quick and accurate ultrasonic measurements, which can provide quick and important information about the condition and quality of the spot welded joint. For high-quality connections, the obtained ultrasonic wave pulse amplitude is in the range 60–98% of the maximum amplitude for the established ultrasonic wave parameters. Considering the research results in the field of the maximum frequency of the spectrum, it should be stated that in stage I, for the value of this parameter in the range of 5.7 to 6.2 MHz, mostly low-quality connections (kissing bond) were obtained. The frequency test results for stage II show that an increase in this frequency above 6.2 MHz indicates kissing bonds. A diagram illustrating the high-quality bond and kissing bond is shown in Figure 9. Moreover, Figure 10 shows selected samples after the destructive test. A close-up of the low-quality (Figure 10a) and high-quality (Figure 10b) welded joints is shown.

Two methods of ultrasonic surface wave propagation in the research presented in the article were used. In the first approach, the heads were placed on one side of the sheets (spot welded joint). In the second approach, they were placed in an alternating pattern. For both approaches, useful signals were obtained on the flaw detector screen for assessing the quality of the spot welded joints. This is an innovative way of testing welded joints. The literature contains research results in the area of technology for making welded joints and their control [34]. The work carried out so far in the field of non-destructive testing of spot welded joints uses a high-frequency longitudinal normal wave [35]. The result of these tests is largely influenced not only by the place where the head is placed (directly above the weld nugget) but also by the pressure force and the direction of the ultrasonic head. Therefore, the results depend largely on the skill and experience of the operator. Other methods of non-destructive evaluation of welded joints are also used, e.g., the passive magnetic flux density testing [18], which, however, requires expensive equipment and strictly defined test conditions. Nevertheless, it should be noted that the proposed approach and the use of an ultrasonic surface wave and its amplitude-frequency spectrum eliminate the above problems that occur in tests using a normal wave ultrasonic head. Therefore, it is an important proposition for people who control such joints, especially when access to the spot weld is difficult.

In the case of ultrasonic testing of steel sheets covered with a zinc coating, the greatest problems arise when the technological requirements of the spot welding process are not respected (too low welding current, too short process time, too little pressure of the electrodes on the steel sheet). If the technological process does not proceed as intended, only the zinc layers may be joined together without the formation of a weld nugget. Then a kissing bond (stick weld) is created. This type of connection is low quality and sometimes difficult to identify during longitudinal ultrasonic wave examination. Moreover, to examine the connection, the head should be installed at the connection point. Using the proposed surface wave testing methodology for spot welded joints, there is no need for direct contact of the heads with the connection. The heads may be at a distance from the joint and the wave traveling along the surface of the metal sheet, hitting the spot welded connection (depending on its quality). This can change the parameters of the ultrasonic wave, which will be important information about its condition. Different positions of spot welded joints on the vehicle body require the operator in factories to apply the longitudinal wave head perpendicularly. The approach proposed in the article, both in stage I and stage II, eliminates difficulties in access to the connection. The surface wave can be sent and received on the same side of the spot welded joint or, if this is not possible, it can be received on the other side of the joint (as shown in stage I).

The advantages and disadvantages of the proposed method for testing spot welded joints over existing and used ultrasonic methods are shown in Table 7.

Qualitative tests of spot welded joints mainly concern the assessment of the weld nugget in terms of its minimum diameter. This type of research is mainly limited to ultrasonic longitudinal wave tests, selecting a transducer with a specific diameter to the required size of the weld nugget. The ultrasonic transducer, depending on its diameter, generates a wave beam of a specific diameter, which passes through the connection area and collects information about its quality. The research presented in this article confirms that the surface wave can also contain indications about the quality of the connection, specifically the weld nugget diameter. In the case of quantitative tests, tests are usually performed on a testing machine to determine the force needed to destroy the welded joint. In the following stage, the remaining weld nugget is assessed through diameter measurements. This type of assessment was also performed as part of this article. The correlation of these two variants of the assessment of spot welded joints is an important novelty and allows for the estimation of quantitative parameters (such as the nugget diameter or the force necessary to destroy the joint—the joint strength) based on the selected parameters of the surface ultrasonic wave pulse.

## 5. Conclusions

This article discusses the important role of spot welded joints in modern automobile construction, emphasizing their significance in strengthening the structural integrity, durability, and overall strength of vehicle bodies. However, sometimes during production stage defects occur, specifically inadequate weld nugget diameters that affect the quality and strength of the connection and the entire structure. Therefore, the article proposes a method for evaluating spot welded joints using ultrasonic surface waves. The study used ultrasonic transducers operating at a frequency of 10 MHz and a dedicated testing setup to analyze a variety of spot welded samples with varying welding process parameters. A key indicator of weld quality investigated in the study is the amplitude of the ultrasonic pulse (pulse height). Another important parameter that allows to assess the quality of a spot weld is the maximum frequency, where the lower the value, the higher the quality of the spot welded connection. In conclusion, the research validates the viability of using surface waves as a reliable means to accurately assess the diameter of the weld nugget in spot welded connections. As directions for further research, the authors intend to determine the values of the maximum frequency response band for specific values of forces destroying the welded joint. Moreover, it is planned to determine the values between two frequency peaks and their changes depending on the quality of the spot welded joint for ultrasonic heads mounted on different sides of the welded joint (stage I). Additionally, a broad analysis for diagnostic features is to be performed, allowing for detailed analysis to draw a relationship between the production parameters and the various features.

## Figures and Tables

**Table 1:** Spot welding process parameters of the samples.

Parameter/Sample	2_1	2_3	3_1	3_6	3_8	3_9	3_10	4_1	4_4	4_5	4_6	4_8
Welding current level kA	2	2	3	3	3	3	3	4	4	4	4	4
Time s	0.15	0.45	0.15	0.9	1.2	1.35	1.5	0.15	0.6	0.75	0.90	1.2

**Table 2:** Comparison of the results of frequency measurements maximum FFT for heads placed on different sides.

No	Measurement Number f_max_					Mean f_max_	SD	L_0.9_
	1	2	3	4	5			
2_1	6.1	5.8	5.8	5.9	5.9	5.9	0.11	0.10
2_3	5.7	5.8	5.9	5.7	5.4	5.7	0.17	0.16
3_1	5.6	5.5	5.7	5.8	5.9	5.7	0.14	0.13
3_6	2.1	2.8	2.5	2.3	2.3	2.4	0.24	0.23
3_8	5.7	5.9	5.6	5.9	5.9	5.8	0.13	0.12
3_9	5.6	6.4	5.7	5.7	5.6	5.8	0.30	0.29
3_10	2.2	2.4	2.4	2.6	2.4	2.4	0.13	0.12
4_1	6.1	5.9	6.3	6.5	6.2	6.2	0.20	0.19
4_4	2.3	2.3	2.6	2.7	2.6	2.5	0.17	0.16
4_5	2.1	2.1	2.4	2.5	2.4	2.3	0.17	0.16
4_6	2.3	2.1	2.1	2.5	2.5	2.3	0.18	0.17
4_8	6.1	6	5.5	5.6	5.8	5.8	0.23	0.22

**Table 3:** Summary of the results of the frequency measurements maximum FFT for heads placed on one side.

No	Measurement Number f_max_					Mean f_max_	SD	L_0.9_
	1	2	3	4	5			
2_1	6.7	6.5	6.6	6.8	6.9	6.7	0.14	0.13
2_3	6.4	6.5	6.7	6.6	6.8	6.6	0.14	0.13
3_1	6.5	6.2	6.1	6.3	6.4	6.3	0.14	0.13
3_6	6.1	6.2	6.1	6.1	6	6.1	0.06	0.06
3_8	6.2	6.2	6.1	6	6	6.1	0.09	0.09
3_9	6.2	6.3	6.1	6.1	6.3	6.2	0.09	0.09
3_10	6.1	6	6.1	6.2	6.1	6.1	0.06	0.06
4_1	6.4	6.2	6.3	6.2	6.4	6.3	0.09	0.09
4_4	5.8	5.9	6	5.9	5.9	5.9	0.06	0.06
4_5	5.9	5.8	6	5.9	5.9	5.9	0.06	0.06
4_6	6	5.9	5.9	6.1	6.1	6	0.09	0.09
4_8	6.2	6.1	6.1	6.1	6	6.1	0.06	0.06

**Table 4:** Summary of the results of pulse height (amplitude) measurements on the flaw detector screen for heads placed on different sides.

No	Measurement Number H (%)					Mean H (%)	SD	L_0.9_
	1	2	3	4	5			
2_1	15	16	13	14	17	15	1.41	1.35
2_3	14	14	12	13	12	13	0.89	0.85
3_1	23	22	21	21	18	21	1.67	1.60
3_6	70	68	71	69	72	70	1.41	1.35
3_8	75	68	69	72	71	71	2.45	2.34
3_9	55	61	55	56	58	57	2.28	2.17
3_10	68	66	68	69	64	67	1.79	1.71
4_1	15	15	17	19	19	17	1.79	1.71
4_4	99	95	93	99	99	97	2.53	2.41
4_5	80	86	87	89	83	85	3.16	3.01
4_6	70	77	73	71	69	72	2.83	2.70
4_8	97	95	88	87	93	92	3.90	3.72

**Table 5:** Summary of the results of pulse height (amplitude) measurements on the flaw detector screen for heads placed on one side.

No	Measurement Number H (%)					Mean H (%)	SD	L_0.9_
	1	2	3	4	5			
2_1	23	26	26	24	26	25	1.26	1.21
2_3	27	26	25	28	29	27	1.41	1.35
3_1	33	36	35	38	33	35	1.90	1.81
3_6	80	76	78	76	80	78	1.79	1.71
3_8	70	70	71	72	72	71	0.89	0.85
3_9	48	46	44	45	42	45	2.00	1.91
3_10	83	80	78	78	81	80	1.90	1.81
4_1	31	34	34	29	27	31	2.76	2.63
4_4	88	87	94	93	88	90	2.90	2.76
4_5	83	81	80	80	81	81	1.10	1.04
4_6	79	82	80	77	77	79	1.90	1.81
4_8	73	74	78	75	75	75	1.67	1.60

**Table 6:** Results of weld nugget destructive test.

Sample	2.1	2.3	3.1	3.6	3.8	3.9	3.10	4.1	4.4	4.5	4.6	4.8
Destructive Force N	957	966	1212	5610	5307	3275	5437	1176	6171	6080	5928	5390
Weld nugget diameter												
X	-	-	-	3.3	2.9	-	3.3	-	3.4	3.4	3.4	2.8
Y	-	-	-	3.5	3.1	-	2.9	-	3.6	3.8	3.4	3.0
Average value	KS	KS	KS	3.4	3.0	KS	2.9	KS	3.5	3.6	3.4	3.1

KS—kissing bond.

**Table 7:** Advantages and disadvantages of testing the spot welded joints by ultrasonic surface wave.

Advantages	Disadvantages
There is no need to apply the ultrasonic head at the spot weld site	Susceptibility of the test result to surface defects in the body sheet metal
There is no need to position the ultrasonic head perpendicular to the welded joint	Research mainly conducted in the frequency domain rather than in the time domain, as in standard research
Does not require testing by an experienced operator	Alignment of the ultrasonic heads during the execution of the test, passing the wave through the spot welded joint
Examination of welded joints that are covered by other components of the vehicle and generation and reception of ultrasonic waves with the help of heads distributed over a long distance	Ensuring constant pressure of ultrasonic heads to the car body sheet metal

## Data Availability

The data presented in this study are available on request from the corresponding author.
